# Issues when the parental and host country systemic institution buffers differ: the case of Czechia

**DOI:** 10.1057/s41261-022-00213-4

**Published:** 2023-01-16

**Authors:** Lukáš Pfeifer

**Affiliations:** Department of Economics and Quantitative Methods, Faculty of Economics, University of West Bohemia and The Czech National Bank, Pilsen, Czech Republic

**Keywords:** Capital buffers, Other systemically important institutions, Subsidiary cap, G2, G18, G21

## Abstract

The article analyses regulatory reforms in the EU to the capital buffers for mitigating risks associated with institutions' systemic importance in the Capital Requirements Directive (CRD). The Directive includes a buffer for other relevant institutions (O-SIIs) and limits its size to a general cap and a specific cap for subsidiaries. However, the specific subsidiary cap may limit national authorities' ability to set a sufficient buffer for domestic institutions that are members of European banking groups to cover risks to the domestic market. It also may lead to a situation where two institutions of similar systemic importance could be subject to different O SII buffer rates because their owners are of different systemic importance and have different O-SII buffer rates in different EU jurisdictions. The amended CRD V increases the general and subsidiary cap for the O-SII reserve by one percentage point. However, the cap for subsidiary institutions remained in force, which limits the setting of capital buffers, especially for banking sectors with significant foreign ownership. These include mainly countries of the former Soviet-aligned Eastern Bloc. This paper outlines shortcomings of the subsidiary cap, argues for a revision of it to ensure level playing field in these capital buffers and quantifies the impact of the subsidiary cap according to the CRD IV and CRD V on the capital requirement applied to the Czech banking sector.

## Introduction

Capital buffers mitigating risks associated with institutions’ systemic importance were introduced into the regulations as part of the reforms responding to the global financial crisis and the problem too big to fail. The buffer for global systemically important institutions (G-SIIs) and the buffer for other systemically important institutions (O-SIIs) are used for this purpose in the regulations in force in the EU. These buffers are designed to mitigate risks associated with the potential destabilisation of systemically important institutions, which could have serious adverse consequences for the financial system and the economy as a whole. The buffers are thus meant to increase the resilience of key banking sector institutions to economic shocks and enable those institutions to continue to provide banking services to the real economy in such situations. However, CRD regulation treats subsidiaries of foreign banks inconsistently compared to parent institutions. This paper examines the O-SII buffer and the implications of the specific approach to subsidiary institutions in this area (Table 1).

**Table 1:** O-SII buffer in the EU regulatory. *Source* CRD IV [5],  CRD V [6]

Capital Requirement Directive (CRD)	Alternative use of the SyRB buffer	The general cap (%)	The subsidiary cap
CRD IV 2014−2021	Yes	2	Higher of 1% or parent institution’s O-SII/G-SII buffer rate
CRD IV 2021−	No	3	1 pp above the parent institution’s O-SII/G-SII buffer rate

*SyRB* Systemic risk buffer

While the general cap is set as a percentage of risk-weighted exposures, the cap for subsidiary institutions is dependent on the level of the parent institution's rate. It may distort the level playing field for domestic O-SIIs, lead to insufficient risk coverage and reduce the predictability of the buffer rate. Institutions designated as O-SIIs accounted for more than two-thirds of EU banking sector assets at the end of 2018 [11] and almost 80% of Czech banking sector assets as of the middle of 2021. The specific subsidiary cap limits national authorities’ ability to set a sufficient buffer for domestic institutions that are members of European banking groups to cover risks to the domestic market. This applies to a greater extent to Central and Eastern European countries, where a larger proportion of O-SIIs was bound by a lower subsidiary cap due to the higher foreign ownership rates of their banking sectors. It also gave rise to a situation where two institutions of similar systemic importance could be subject to different O SII buffer rates because their owners are of different systemic importance and have different O-SII buffer rates in different EU jurisdictions.

On the other hand, there are also arguments used to justify having a specific subsidiary cap derived from the parent’s O-SII/G-SII buffer rate. One such argument is that it is more effective for institutions that are members of large groups with high sectoral and geographical diversification to manage their risks centrally, because they are less prone to shocks than domestically owned banks and have access to support from their parent banks [11]. On the other hand, Cull et al. [8] state that the experience of the global financial crisis shows that foreign parent institutions can, on the contrary, amplify shocks. This may be particularly relevant in the event of a synchronous shock across EU economies. Furthermore, D'Hulster [7] states that the global financial crisis has confirmed that individual supervisors ignore the impact of their decisions on financial stability in other jurisdictions and has also highlighted the systemic risks host supervisors whose financial systems are dominated by foreign banks face. Singh [19] analyses how international standards and EU requirements undertake to divide responsibilities between the home and host state and the extent to which they align interests between the home and host and minimise potential conflicts of interests.^1^

This introduction is followed by Section II describing the main causes of the different approaches used to mitigate risks associated with institutions’ systemic importance applied in the EU – in the Capital Requirement Directive (hence on, CRD). Section III describes the legislative changes in this area contained in CRD V,^2^ which, together with a revision of the EBA methodology setting a floor for the buffer rate, should reduce these differences. Section IV describes implications of regulatory changes for the level of capital buffers in the Czech banking sector. Section V summarises and concludes.

## Approaches to mitigating risks associated with institutions’ systemic importance in the EU before the transposition of CRD V

The approaches used to mitigate risks associated with the systemic importance of institutions have differed considerably across the EU to date. This heterogeneity in setting a buffer to limit the risks associated with systemic importance is not clearly explained by differences in the institutions concerned, the ratio of assets to GDP, or by the Member State’s position in the financial cycle [11, 15]. It is due to differences in legislation concerning the O-SII cap (see Sect. "The effect of the subsidiary cap on the O-SII buffer") and differences in the approaches used to set buffer rates (see Sect. "The effect of the method used to set the O-SII buffer rate") depending on institutions’ level of systemic importance.

### The effect of the subsidiary cap on the O-SII buffer

The CRD IV regulations in force in the EU, and hence also in the Czech Republic, from 2014 until 2021 allowed authorities to set an O-SII buffer rate of up to 2% institution’s risk-weighted exposures. For systemically important institutions that are subsidiaries of European parent institutions designated by their home supervisory authorities as O-SIIs or G-SIIs, this cap was set at the parent institution’s O-SII buffer rate or 1%, whichever was the highest (Article 131(8) of CRD IV).^3^

**Fig. 1:**
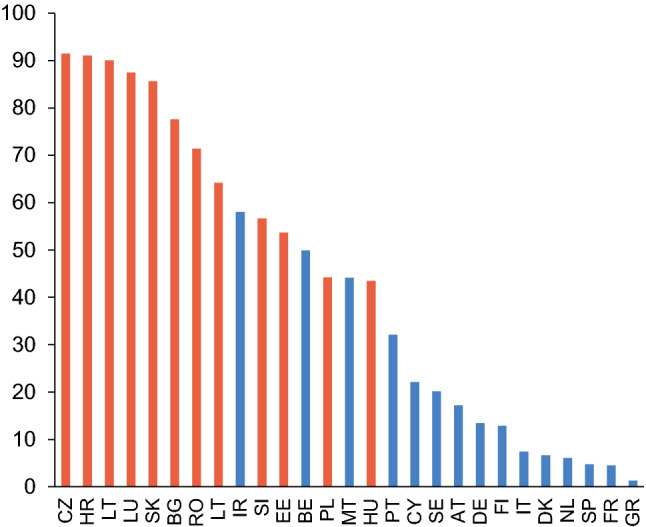
Foreign ownership rates of banking sectors in the EU. (in % as of 2020 Q2). *Source* ECB. Central and Eastern European countries are indicated in red

The subsidiary cap limits national authorities’ ability to set a sufficient buffer for domestic institutions that are members of European banking groups to cover risks to the domestic market, because the cap applied to such institutions may be lower than the general limit. ESRB [15] states that 30 O-SIIs were bound by the subsidiary cap and were therefore subject to a rate of less than 2%.^4^ This applies to a greater extent to Central and Eastern European countries, where a larger proportion of O-SIIs was bound by a lower subsidiary cap due to the higher foreign ownership rates of their banking sectors (see Fig. 1). In some EU Member States, including the Czech Republic, this subsidiary cap was therefore assessed as insufficient to cover the risks identified [20]. It also gave rise to a situation where two institutions of similar systemic importance can be subject to different O-SII buffer rates because their owners are of different systemic importance and have different O-SII buffer rates in different EU jurisdictions. This situation is not in conformity with the BCBS framework for dealing with domestic systemically important banks [2], according to which authorities should treat banks equally when setting the buffer regardless of the characteristics of their owners. Otherwise, risks are not covered sufficiently and the level playing field principle is compromised.

Linking the cap to the nature of the institution’s owner also implies potential volatility of this capital buffer in the event of an increase in acquisition and consolidation processes in the domestic and European banking sector.^5^ Another potentially sensitive issue is O-SII buffer rate volatility resulting from macroprudential authorities changing the buffer rates in parent banks’ home countries. This may make the playing field less level in some cases and potentially also make it more difficult for institutions to plan their capital. During the coronavirus crisis, several Member States (Cyprus, Finland, Hungary, the Netherlands) lowered their buffer rates to zero [18] to give institutions more capital room for lending,^6^ even though their systemic importance scores had not decreased. This practical experience indicates that it is appropriate to continue to debate the relevance of having a separate subsidiary cap.

There is some argument for subsidiary cap arises from the fact that the O-SII buffer is set on the basis of consolidated data.^7^ Nonetheless, consolidated data only capture the subsidiary’s relative importance in the group. So, while the subsidiary may be of very little importance in the group (i.e. have low systemic importance risk for the parent), it may be a dominant institution in the Member State where it operates (i.e. have greater systemic importance risk in its home country; see Table 2).

**Table 2:** Importance of domestic O-SIIs in their groups in 2020. *Source* CNB, ESRB, author’s calculations

Subsidiary	Systemic importance score	SRB rate applied previously	Parent institution	Systemic importance score	O-SII/G-SII buffer rate	Parent-subsidiary exposure ratio
ČSOB	22.5	3.0	KBC Group (BE)	23.5	1.5	24.9
ČS	15.6	3.0	Erste Group (AT)	25.2	2.0	22.3
KB	15.1	3.0	Société Générale (FR)	18.5	1.0	3.9
UCB	11.0	2.0	UniCredit (IT)	30.1	1.0	3.4
RF	6.7	1.0	Raiffeisen Bank (AT)	14.6	2.0	8.4

Systemic importance score is assessed on the basis of the harmonised EBA Guidelines [10]. *ČSOB*  Československá obchodní banka; *ČS *Česká spořitelna; *KB* Komerční banka; *UCB* UniCredit Bank Czech Republic and Slovakia; *RF* Raiffeisenbank

Given the constrained cap on the O-SII buffer rate and the key role of domestic O-SIIs in financing the economy, many Member States, among them the Czech Republic [20], therefore exercised other legislative options to cover the identified level of risk associated with systemically important institutions. Eleven EEA Member States (Austria, Czech Republic, Denmark, Finland, Croatia, Liechtenstein, the Netherlands, Norway, Sweden, Slovakai, United Kingdom) used the SyRB [15].^8^ Sweden and the Netherlands, for example, applied an SyRB rate of 3% to total exposures alongside the O-SII buffer. Under CRD IV (Article 131(15)), however, the SyRB was not cumulative with the O-SII buffer when applied to total exposures, so the higher of the two applied. The 3% SyRB rate was thus binding regardless of the O-SII buffer rate. Some Member States (such as Slovakai) set an SyRB solely for domestic exposures, in which case it was cumulative with the O-SII buffer. Instead of the O-SII buffer, Czech Republic, United Kingdom and Denmark used the SyRB only. This allowed them to set a higher cap to cover the risk associated with the systemic importance of institutions.

### The effect of the method used to set the O-SII buffer rate

The process of setting the O-SII buffer rate starts with the identification of systemically important institutions. Systemic importance is assessed on the basis of the harmonised EBA Guidelines [10] using a scoring system. Institutions are scored according to several indicators describing four key characteristics: (i) size, (ii) substitutability, (iii) cross-border activity and (iv) interconnectedness. The Guidelines also allow the use of optional indicators and supervisory judgement. This enables differences across banking sectors and institutions to be taken into account while preserving some degree of discretion in the O-SII identification process. EBA [11] states that 29 institutions (out of a total of 196 O-SIIs) were designated as O-SIIs in 2020 on the basis of additional supervisory assessments.

In the next step, the O-SII buffer rate is set. There are no harmonised guidelines for this part of the process; this is so that sufficient room is left for macroprudential authorities to reflect the specificities of each national banking sector and institution. EU Member States thus use various methods to set the O-SII buffer rate, although most of them work with methods based on directly assigning the O-SII buffer rate according to the systemic importance score determined in the O-SII identification process. The most common method is the bucketing approach, which divides scores into several buckets and assigns an O-SII buffer rate to each bucket. The higher the bucket, the higher the rate. A much smaller set of countries use methods that involve no direct link between the rate and the score.^9^ These include the equal expected impact (EEI) approach [20] and the use of stress tests, where the aim is to set the rate so that the impact of the failure of a systemically important institution equals that of the failure of non-systemically important institutions. Combinations of these methods are also used. Each incorporates some degree of expert supervisory judgement where the quantitative approach is complemented by a broader qualitative holistic assessment of the general characteristics of the relevant banking system and the institution’s systemic role in it. In the most frequently used bucketing approach, expert judgement is used mainly in setting the number of buckets, the bucket bandwidths and the cap in the highest bucket. The number of buckets ranges from three (Austria) to twelve (Germany). The absolute caps and the rates assigned to the intervals of scores also differ [15]. In 2020, a total of 36 European institutions were subject to an O-SII buffer rate of 0.5%, while 49 were subject to a rate of 1% and 37 to a rate of 2%. Some countries opt for a generally more cautious approach to setting the buffer rate than others. In Italy and Sweden, for instance, a systemic importance score of 2,500 bp implies an O-SII buffer rate of 0.75%–1%, whereas in countries such as France, Liechtenstein and Latvia it implies a rate of 1.5%–2% [16]. Austria had the lowest score corresponding to a 2% rate (1,000 bp), while Slovenia had the highest (5,250 bp). In Italy and Spain, for example, the maximum O-SII buffer rate is lower than the legislative cap of 2%. Overall, then, we can say that the use of different methods with different emphases on the expert judgement in setting the O-SII buffer rate contributed to the differences in O-SII buffer rates across the EU Member States.

At the end of the rate-setting process, the national macroprudential authority notifies the relevant EU bodies of the rate and explains the designation of institutions as O-SIIs and the calibration of the rate with regard to the risks identified. This ensures that the entire process is transparent across EU countries. The list of O-SIIs in the relevant country and the O-SII buffer rates they are subject to are then published on the websites of the national macroprudential authorities and the ESRB.^10^

## Amendments to the S_y_RB and O-SII buffer rules

In the review of macroprudential policy in the EU, the differences in the design and calibration of O-SII buffer rates in past years were viewed as potentially compromising the level playing field [11, 14]. As a result, legislative changes were made to the macroprudential framework in the structural buffers area in CRD V. The EBA floor methodology for O-SII buffer rates was also revised.

The changes contained in CRD V mean that authorities can only use the O-SII buffer–and hence not the SyRB–to mitigate risks associated with the systemic importance of institutions. They will be still able to apply the SyRB to cover other structural risks, and now in a more flexible way. Under CRD IV the SyRB could only be applied universally either to all exposures or to all domestic exposures, whereas CRD V allows it to be applied to subsets of sectoral exposures as well [12]. It will also be possible to use the SyRB to cover cyclical risks that cannot be mitigated with the countercyclical capital buffer (CCyB).

The O-SII buffer rate is now capped at 3%.^11^ In the case of domestic banks that are subsidiaries of foreign institutions designated by their home supervisors as O-SIIs or G-SIIs, the O-SII buffer cap may be no more than 1 pp above the foreign institution’s O-SII/G-SII buffer rate.^12^ In addition, the “cumulativeness” of the O-SII, G-SII and systemic risk buffers has been simplified. Where the G-SII and O-SII buffers overlap, the higher of the two becomes binding. If the institution is simultaneously subject to the SRB, it is cumulative with the O-SII buffer or the G-SII buffer. However, the sum of all these buffers should not exceed 5% unless authorisation has been obtained from the Commission.^13^,^14^

The next step towards increased harmonisation and convergence of the approaches applied by EU Member States was the specification of a non-binding floor methodology for O-SII buffer rates. Originally created in 2016 for the banking union member countries, it used systemic importance scores determined in accordance with the EBA Guidelines combined with the bucketing approach. In 2020 the EBA revised the methodology [11] and recommended that it be applied by all the EU Member States as from 2022. The methodology defines four buckets, each associated with a specific O-SII buffer rate floor (see Table 3). Under the new methodology, six Member States would see at least one O-SII with a higher buffer rate than at present.^15^

**Table 3:** The EBA’s bucketing approach to calibrating the minimum O-SII buffer rate. (in %). *Source* EBA [11]

Bucket	Score	Rate (%)
1	≤ 1.249	0.25
2	1250–1949	0.50
3	1950–2899	0.75
4	≥ 2900	1.00

## The Czech approach to mitigating risks associated with the systemic importance of institutions

### Before the transposition of CRD V: 2014–2021

Five other systemically important institutions (ČS, ČSOB, KB, UCB, RB) were identified in the Czech banking sector for 2021 (before the transposition of CRD V).^16^ These institutions were subject to an SyRB of 1%–3% depending on their level of domestic systemic importance. The SyRB buffer rate was set on the basis of an assessment of each bank’s domestic systemic importance using a range of indicators describing four key parameters of the bank: size, substitutability, cross-border activity and interconnectedness [20]. The calculation of the domestic systemic importance of institutions was based principally on the EBA methodology but differs in some of the indicators used. The main methodological difference, however, was that the EBA methodology for O-SIIs works primarily with data for consolidated groups containing both bank and (selected) non-bank entities, including those in foreign ownership, whereas the Czech National Bank (hence on, CNB) methodology used data for banks on an individual basis to set the SyRB rate.

All the institutions subject to a nonzero SyRB rate are subsidiaries of foreign banks designated by their home supervisors as O-SIIs or G-SIIs (see Table 2). The parent bank’s O-SII/G-SII buffer rate therefore affected the O-SII buffer cap that could be applied to the relevant domestic O-SIIs. Table 4 illustrates the impact of this. It is apparent that if the O-SII buffer had been applied, four of the five domestic O-SIIs would have been subject to a lower rate, and hence a smaller buffer, than in the case of the SyRB rate actually applied. The buffer mitigating risks associated with systemic importance would thus have shrunk by a sizeable CZK 24 billion to 53% of the SyRB level.

**Table 4:** SyRB buffer rates for O-SIIs and legislative options under CRD IV. (in % unless otherwise indicated; as of 2021 Q2). *Source* CNB, author’s calculations

Institution	Systemic importance score	SRB rate before transposition of CRD V	Parent bank’s O-SII/ G-SII buffer rate	O-SII buffer cap under CRD IV	Difference w.r.t. SRB rate	Difference w.r.t. SRB rate (in CZK billions)
ČSOB	22.5	3.0	1.5	1.5	– 1.5	– 6.1
ČS	15.6	3.0	2.0	2.0	– 1.0	– 5.4
KB	15.1	3.0	1.0	1.0	– 2.0	– 9.3
UCB	11.0	2.0	1.0	1.0	– 1.0	– 3.6
RB	6.7	1.0	2.0	2.0	1.0	2.0

Systemic importance score is assessed on the basis of the harmonised EBA Guidelines [10]. *ČSOB* Československá obchodní banka; *ČS* Česká spořitelna; *KB* Komerční banka; *UCB* UniCredit Bank Czech Republic and Slovakia; *RF* Raiffeisenbank

### After the transposition of CRD V: the present

Since the transposition took effect on 1 October 2021, the CNB has only been able to use the O-SII buffer to mitigate risks associated with systemic importance. To identify O-SIIs, it continues to use systemic importance scores determined at the consolidated level in accordance with the EBA Guidelines (2014).

To determine the O-SII buffer rate the CNB apply the bucketing approach, with each interval of systemic importance scores associated with a specific buffer rate. The bucketing approach is the method most commonly applied by the EU Member States (17 countries) and is also used at the international level (the EBA O-SII floor methodology and the G-SII buffer rate-setting methodology).^17^ The CNB has chosen an O-SII buffer cap of 3% as the highest rate in the highest bucket under CRD V (Table 4).^18^ This is equal to the highest SyRB rate that it previously used to mitigate risks of systemic importance and thus ensures continuity, consistency and predictability of the CNB’s macroprudential policy in this area. The use of six buckets with the same bandwidths (300 bp) allows the CNB to appropriately link different levels of systemic importance to the buffer rate. The methodological transparency of the process of identifying institutions and setting O-SII buffer rates will help institutions to manage their capital effectively. An annual review of the list of O-SIIs and the buffer rates enables the CNB to take account of changes within institutions and banking sector (such as changes to business policies, and mergers and acquisitions) (Table 5).^19^

**Table 5:** The CNB’s bucketing approach to setting the O-SII buffer rate. (in %). *Source* CNB

Bucket	Score	Rate (%)
1	425–724	0.5
2	725–1024	1.0
3	1025–1324	1.5
4	1325–1624	2.0
5	1625–1924	2.5
6	≥ 1925	3.0

The CNB is switching from its previous SyRB rate-setting approach based on assessing the systemic importance of individual banks [20] to assessing systemic importance at the institution’s highest tier of regulatory consolidation at the Czech Republic level. Once the CNB has determined the O-SII buffer rate, it will compare it with the regulatory O-SII cap. In the case of domestic banks that are subsidiaries of foreign banks designated by their home supervisors as O-SIIs or G-SIIs, it will adjust the O-SII cap so that it satisfies the limit of 1 pp above the foreign bank’s O-SII/G-SII buffer rate. In the next step, the CNB will conduct a supervisory assessment, in which it will take into account any specificities of the institution in the final calibration of the O-SII buffer rate in particular the risks associated with its activities in third countries. Table 6 shows–for each O-SII–the rate set under the bucketing approach, the rate adjusted in accordance with the legislative cap, and the rate applied after supervisory assessment.

**Table 6:** Calibration of the O-SII buffers of domestic institutions. (in % unless otherwise indicated; as of 2021 Q2) *Source* CNB, author’s calculations

Institution	Systemic importance score	SRB rate before transposition of CRD V	O-SII buffer rate under bucketing approach	O-SII buffer rate taking CRD V cap into account	O-SII buffer rate after supervisory assessment (rate applied)	Difference w.r.t. SRB rate (in pp)	Difference w.r.t. SRB rate (in CZK billions)
ČSOB	22.5	3	3	2.5	2.5	– 0.5	– 2.0
ČS	15.6	3	2	2	2	– 1.0	– 5.5
KB	15.1	3	2	2	2	– 1.0	– 4.7
UCB	11.0	2	1.5	1.5	1.5	– 0.5	– 1.8
RB	6.7	1	0.5	0.5	0.5	– 0.5	1.0

Systemic importance score is assessed on the basis of the harmonised EBA Guidelines [10]. *ČSOB* Československá obchodní banka; *ČS* Česká spořitelna; *KB* Komerční banka; *UCB* UniCredit Bank Czech Republic and Slovakia; *RF* Raiffeisenbank

The bucketing approach calibration on 2021 Q2 data lead to a decline in capital of around CZK 13 billion (25% of the previously applied SRB) relative to the SyRB maintained before the transposition of CRD V. Following the bucketing approach calibration, the O-SII buffer rate is 1 pp lower for ČS and KB and 0.5 pp lower for UCB and RB compared with the previously applied SRB. The constraint due to the parent institution’s O-SII buffer rate would lead ČSOB’s rate to decrease from 3% under the bucketing approach to 2.5% (KBC is subject to an O-SII buffer rate of 1.5%). The constraint due to the parent institution’s O-SII buffer rate alone would thus cause its O-SII buffer to fall by CZK 2 billion (4% of the previously applied SyRB).^20^ After the overall assessment, the O-SII buffer would therefore be CZK 15 billion lower than the previously applied SyRB (29% of the total previously applied SyRB).

However, the decline in the buffer should be viewed not only in the context of the total capitalisation of the banking system, but also from the perspective of the availability of funds enabling systemically important institutions to be recapitalised (the minimum requirement for own funds and eligible liabilities, hence on, MREL).^21^ The MREL, which is designed to provide for the resolution of institutions and ensure the continuity of critical functions in the banking sector, has a similar goal as the O-SII buffer, namely to mitigate the risk arising from the destabilisation of institutions that are important to the national banking sector (for details, see [18]. As a result, the decrease in the capital requirement resulting from the lower O-SII buffer relative to the existing SyRB should not lead to a significant reduction in the resilience and financial stability of systemically important institutions.

## Conclusion

This article presented the main reasons for the different approaches used to mitigate risks associated with institutions’ systemic importance applied in the EU Member States when CRD IV was in force (2014–2021). Those reasons included a low O-SII cap for subsidiaries of foreign companies and the use of different methodological approaches to set buffer rates. The article also described legislative changes arising from CRD V which provide for further harmonisation of the EU Member States’ approaches in this area. These include a requirement for authorities to use only the buffer for systemically important institutions to mitigate risks associated with the systemic importance of institutions. The O-SII cap that may be applied without authorisation from the Commission has been raised by 1 pp to 3%. On the other hand the CRD V remains in force specific subsidiary cap derived from the parent’s O-SII/G-SII buffer rate.

The article quantifies the differences in the capital requirement when the O-SII buffer is used according to the caps set through Directive CRD IV and CRD V in the Czech banking sector. If the O-SII buffer had been applied during the validity of CRD IV, four of the five domestic O-SIIs would have been subject to a lower rate, and hence a smaller buffer, than in the case of the SyRB rate actually applied. The buffer mitigating risks associated with systemic importance would thus have shrunk by a sizeable CZK 24 billion to 53% of the SyRB level. After the transposition of CRD V, the gap has narrowed. Using the 2021 Q2 data, the CNB's new approach lead to a decrease in the buffer mitigating risks associated with systemic importance by around CZK 15 billion, or 29% of the previously applied SyRB. This is mainly because of a reduction in the buffer rates applied to some institutions, resulting from more sensitive differentiation of the degree of systemic importance than under the SyRB rate-setting approach [20]. In addition, the O-SII buffer rate is reduced in one institution by the constraint arising from the parent institution's O-SII buffer rate. The inability to use the systemic risk buffer to mitigate the risks associated with the systemic importance of institutions, together with the existence of the subsidiary cap, led to a reduction in the associated capital buffer despite the increase in the O-SII buffer cap following the transposition of CRD V into Czech law.

In general, it can be stated that, the specific subsidiary cap limits the room for manoeuvre of macroprudential authorities in terms of capital buffers limiting systemically important risks for countries with significant foreign ownership of the banking sector, which are mostly countries of the former Soviet-aligned Eastern Bloc. Therefore, the persisting legislative constraint where the subsidiary cap is derived from the parent company’s rate may distort the level playing field for domestic O-SIIs and reduce the predictability of the buffer rate, especially in the event of unexpected economic developments linked with a reduction of the parent institution’s O-SII buffer rate to zero or in the event of changes in ownership structure.

## Footnotes

1 World Bank [21] describes the possibilities for host authorities to improve their negotiating position in the EU supervision and resolution area.

2 CRD IV is the CRD update to the European Banking System legislated in 2013. CRD V is the update legislated in 2019.

3 A maximum buffer rate of 1% could be set where the parent group’s buffer rate was lower than 1%.

4 Of the total of 187 EuropeanG/O-SIIs identified, 56 were subsidiaries.

5 This is one way to mitigate the risk of low banking sector profitability[1], 15. On the other hand, it can create risks of lower competition and higher prices for consumers [11].

6 A macroprudential authority may reduce the countercyclical buffer rate to zero in response to cyclical developments in the economy (Article 136(6) ofCRD IV; for more details, see [19]. However, the buffer limiting risks associated with systemically important institutions should not be released and should only be used to absorb losses or lend to the economy as the last of the capital buffers [6].

7 Buch and Goldberg [4] state prudential instruments spill over across borders through international bank lending.

8 SyRB can be used to suppress various sources of structural risks to banking sector stability except for the risk associated with the systemic importance of the institution, for which the O-SII buffer is used after the transposition of CRD V. For more details on the interaction of structural buffers, see ESRB [16].

9 O-SIIs’ scores still have to be updated every year.

10 For an overview of capital buffers in the EU, seehttps://www.esrb.europa.eu/national_policy/systemically/html/index.en.html.

11 This is 1 pp higher than the CRD IV cap. A higher rate may be imposed subject to approval from the Commission.

12 Specifically, the lower of 3% and the foreign institution’s O-SII/G-SII buffer rate + 1% (or the rate applied to the parent institution if it is higher than 3%). This is 1 pp higher than the CRD IV cap.

13 The caps on the O-SII buffer rate alone and on the O-SII buffer rate cumulative with the SyRB may only be exceeded with authorisation from the Commission.

14 Furthermore, unlike other coordination limits in other capital buffer provisions, the cap for subsidiaries cannot be overridden in justified cases. Please note that the 3% threshold for O-SII buffer rates, 3% and 5% thresholds for systemic risk buffer (hence on, SyRB) rates, does not prevent authorities to go beyond, subject to EU coordination procedure. The hard nature of the cap for subsidiaries not just creates obstacles for macroprudential policy in some countries and for the single market, but also creates logical incontinency in the framework. It is not clear, for example, why, theoretically, a 5% O-SII buffer on a domestic bank should be possible, subject to an EU authorization, whereas a 2% buffer on a subsidiary of a parent with 1% O-SII buffer rate is not permitted.

15 According to an EBA questionnaire based on end-2019 data

16 In accordance with the EBA methodology: https://www.cnb.cz/en/financial-stability/macroprudential-policy/list-of-other-systemically-important-institutions/.

17 BCBS [3] proposes a similar system for the calibration of G-SII buffers.

18 Past and present CNB analyses indicate that the O-SII cap needs to be at least 3% to cover risks associated with systemic importance [20].

19 The CNB evaluated alternative rate-setting approaches that would reduce the impact of the switch from the SyRB to the O-SII buffer on the total size of the buffer. However, these approaches would create greater distortions of the level playing field.

20 The supervisory assessment does not have any fundamental effect on the level of systemic importance of the institutions previously identifiedas O-SIIs (see Table 6.

21 An intermediate objective has been in effect since 1 January 2022 and must be fully met by 1 January 2024 in the Czech Republic. The MREL is designed to ensure that banks have sufficient capacity for the absorption of losses and subsequent recapitalisation in the event of resolution. The MREL can be met using capital, eligible liabilities or a combination thereof.
